# Mutational basis of ceftazidime-borrelidin A collateral sensitivity in *Escherichia coli*

**DOI:** 10.1093/g3journal/jkag046

**Published:** 2026-03-04

**Authors:** Laura L Phillips, Arianna Carrasco, Jonathan Weiss, Dennis Y Liu, Roger G Linington, Alex Wong

**Affiliations:** Department of Biology, Carleton University, 1125 Colonel By Dr., Ottawa, ON K1S 5B6, Canada; Institute for Advancing Health Through Agriculture, Texas A&M AgriLife, 1500 Research Parkway, College Station, TX 77845, United States; Institute for Advancing Health Through Agriculture, Texas A&M AgriLife, 1500 Research Parkway, College Station, TX 77845, United States; Department of Chemistry, Simon Fraser University, 8888 University Dr., Burnaby, BC V5A 1S6, Canada; Department of Chemistry, Simon Fraser University, 8888 University Dr., Burnaby, BC V5A 1S6, Canada; Department of Biology, Carleton University, 1125 Colonel By Dr., Ottawa, ON K1S 5B6, Canada; Institute for Advancing Health Through Agriculture, Texas A&M AgriLife, 1500 Research Parkway, College Station, TX 77845, United States

**Keywords:** *Escherichia coli*, antibiotic resistance, collateral sensitivity, ceftazidime, borrelidin

## Abstract

The rapid emergence of antimicrobial resistance in bacterial pathogens threatens the efficacy of nearly all available antibiotics. One evolution-informed strategy to limit the emergence of resistance is the exploitation of collateral sensitivity, whereby resistance to one compound results in increased sensitivity to another. Here, we investigate the collateral sensitivity relationship between the cephalosporin antibiotic ceftazidime and the natural product borrelidin A in *Escherichia coli*. Previously, we found that borrelidin A prevented the evolution of clinical ceftazidime resistance during laboratory selection. In this study, we further characterized the genomic and phenotypic consequences of evolution under collateral sensitivity in these evolved populations. Co-dosing with 128 µM borrelidin A significantly reduced the evolution of ceftazidime resistance, while preserving fitness in the absence of drug. Overall co-dosed strains had reduced resistance and cross-resistance to all tested antibiotics. Whole-genome sequencing revealed that co-dosing suppressed the accumulation of single nucleotide polymorphism and insertion/deletion mutations in known resistance-associated genes yet resulted in an increased number of mobile-element insertion mutations. In one case, we find a possible novel borrelidin A resistance mutation. Our results suggest that ceftazidime-borrelidin A co-dosing limits both resistance and cross-resistance through selection against costly resistance mutations. While borrelidin A itself is cytotoxic, our findings highlight the promise of targeting bacteria-specific vulnerabilities to curb the emergence of multidrug resistance. These findings contribute to the growing body of evidence supporting collateral sensitivity-informed approaches as practical strategies to mitigate antimicrobial resistance in bacterial populations.

## Introduction

Antibiotics were a major medical breakthrough of the 20th century (Katz and Baltz 2016), but their effectiveness has been undermined by the emergence of antimicrobial resistance (AMR), posing a critical threat to global public health (Davies and Davies 2010). In response to this growing crisis, the World Health Organization updated its Bacterial Priority Pathogen List in 2024, identifying 24 pathogens across 15 families posing as high-risk global threats with significant economic burdens (WHO bacterial priority pathogens list 2024). Among the critically classified group of priority pathogens are the carbapenem and third-generation cephalosporin-resistant *Enterobacterales*, including *Escherichia coli*, which are responsible for the greatest proportion of AMR-associated deaths (Murray et al. 2022; WHO bacterial priority pathogens list 2024).

The evolution of AMR is a predictable consequence of antimicrobial use, with the simple use of antibiotics driving resistance (Larsson and Flach 2022; Muteeb et al. 2023). As a result, nearly every antibiotic on the market today already has known associated resistances (Zhang et al. 2022; Muteeb et al. 2023). It is therefore important to understand how antibiotic use influences the many routes to resistance.

Evolved resistance to one antibiotic may further result in cross-resistance (CR) (Uddin et al. 2021). CR occurs when resistance to one antibiotic leads to resistance to other drugs, even without exposure to the secondary drug (Uddin et al. 2021). Because many genes and mutations confer resistance to multiple drugs, CR further exacerbates the threat posed by drug and multidrug-resistant (MDR) pathogens (Lázár et al. 2014). Overall, infections caused by MDR pathogens are associated with higher mortality rates, prolonged hospitalizations, and increased economic burdens (Maragakis et al. 2008; van Hecke et al. 2017).

One approach to tackling resistance is to exploit vulnerabilities specific to AMR pathogens. While resistance toward a particular drug is obviously beneficial for a pathogen in the presence of that drug, it is often accompanied by one or more trade-offs, such as reduced fitness in the absence of drug (Melnyk et al. 2015; Vogwill and MacLean 2015), or increased sensitivities to additional molecules (Pluchino et al. 2012; Lázár et al. 2013; Pál et al. 2015). Such resistance-associated sensitivity is known as negative resistance, or more commonly, collateral sensitivity (CS) (Lázár et al. 2013, 2018; Pál et al. 2015). CS interactions have been widely observed in drug-resistant pathogens and can slow, or even reverse, the evolution of resistance through the use of pairs of drugs that are mutually collaterally sensitive, either in combination or cyclically (Imamovic and Sommer 2013; Liu et al. 2023; Maltas et al. 2025). CS has also been observed between antibiotic resistance and nonantibiotic stressors (Maltas et al. 2020).

Several studies to date have mapped CS interactions between known antibiotics for key pathogens including *Pseudomonas aeruginosa* (Hernando-Amado et al. 2020), *Enterococcus faecalis* (Maltas et al. 2025), and *E. coli* (Imamovic and Sommer 2013; Liu et al. 2023; Herencias et al. 2024; Sakenova et al. 2025). The mechanisms underlying CS have been described in several cases (Lázár et al. 2013, 2018; Rosenkilde et al. 2019; Roemhild et al. 2020), but further work remains.

We previously investigated CS relationships using resistant strains of *E. coli* and a library of over 6000 natural product (NP) extract prefractions, derived from marine Actinobacteria and terrestrial *Burkholderia* (Liu et al. 2023). We found that strains harboring ceftazidime (Cef) resistance mutations in *rfaG* (also known as *waaG*) and *rfaH* showed increased sensitivity to the known natural product borrelidin A—i.e. ceftazidime resistance was accompanied by CS to borrelidin A (Liu et al. 2023). Laboratory selection of susceptible populations in the presence of Cef and borrelidin A suppressed the evolution of ceftazidime resistance, while populations dosed with ceftazidime alone rapidly achieved clinically relevant levels of resistance (Liu et al. 2023).

Here, we further investigate the suppression of the evolution of ceftazidime resistance by borrelidin A. Given the susceptibility of several Cef^R^ mutants to borrelidin A in our original screen (Liu et al. 2023), we hypothesize that borrelidin A selects against Cef^R^ mutants. We further investigate the impact of ceftazidime and borrelidin A co-dosing on CR, CS, and fitness. Our work ultimately serves to further our understanding of the complex nature of the evolution of antibiotic resistance in the context of CS. Leveraging CS as a method to prevent the evolution of drug and multidrug resistance may help alleviate the challenges of antibiotic resistance, as well as offer potential strategies to restore the efficacy of existing antibiotics, mitigating the challenges currently posed by MDR pathogens.

## Methods

### Bacterial strains and growth conditions

All experiments were carried out using *E. coli* K-12 (MG1655; Yale *E. coli* stock center) or derivatives thereof. Bacteria were routinely cultured at 37 °C in lysogeny broth (Miller) (LB; 10 g/L tryptone, 5 g/L yeast extract, 10 g/L NaCl), with shaking (150 rpm) or plated on LB with 1.5% agar (LBA).

A *ΔlacIZYA* strain (Lac−; AH171) was constructed from MG1655 for use in blue/white competition screening, following Hinz et al. (2024). This knockout was constructed using a custom allelic replacement (AR) suicide plasmid (pR6KT-SacB-*ΔlacIZYA*) (Lebeuf-Taylor et al. 2019; Hinz et al. 2022), replicating only in hosts carrying the *λpir* gene (Rakowski and Filutowicz 2013; Hinz et al. 2024). Selectable (tetracycline resistance) and counter-selectable (*sacB*) markers on the plasmid facilitated 2-step allelic exchange between the plasmid and the bacterial chromosomal sequence (Hmelo et al. 2015; Hinz et al. 2024). The *lac* targeting sequences were PCR-amplified from the MG1655 chromosomal DNA using the primers in Table 1 and ligated to the pR6KT-*sacB* plasmid by 1-pot Golden Gate assembly reaction containing the type IIS restriction enzyme Bsal and T4 DNA ligase, as described in Hinz et al. (2022). Assembly reactions were transformed into chemically competent *E. coli* DHα *λpir* by the Inoue method (Sambrook 2001).

**Table 1.** Oligo primer sequences for Golden Gate assembly and allelic replacement for *ΔlacIZYA* AH171 construction.

Oligo	Sequence (5′ to 3′)	Amplicon description
mhpR-F	ACTGCGGGTCTCAgtcgCCTGCATCGCACCACTGT	723 bp upstream from *lacI*
mhpR-R	ACTGCGGGTCTCAtgggGCCATTCGATGGTGTCAAC	723 bp upstream from *lacI*
cynX-F-lac	ACTGCGGGTCTCAcccaTAAACGACCGGGATAAGCAC	774 bp downstream from *lacA*
cynX-R	ACTGCGGGTCTCAgcaaTTCCCACAAGACAACAACCA	774 bp downstream from *lacA*

Primers amplified *E. coli* sequences upstream and downstream of the *lac* operon. Golden Gate assembly of the amplicons to the pR6KT-*sacB* allelic replacement vector utilized BsaI enzyme recognition sequences (underlined) and 4 bp overhangs (lowercase) as described in Hinz et al. (2022).

The AR plasmid was transformed into the diaminopimelic acid (DAP) auxotrophic donor strain WM3064, a *pir*-expressing *E. coli*, and subsequently conjugated from the donor to the recipient MG1655, with selections for homologous recombination between plasmid and chromosomal sequences following Hinz et al. (2024).

### Laboratory selection

Populations of *E. coli* evolved in ceftazidime with or without borrelidin A were reported in Liu et al. (2023). Each population was started with an independent colony of *E. coli* K-12 (MG1655), in triplicate per treatment, under 4 treatments: (i) CEF treatment, increasing concentrations of ceftazidime starting at 0.063 µM (see below for details); (ii) CEFBOR32 treatment, CEF treatment plus a constant concentration of borrelidin A at 32 µM; (iii) CEFBOR128 treatment, CEF treatment plus a constant concentration of borrelidin A at 128 µM; and (iv) a CEFNOR treatment, CEF treatment with additional increasing concentrations of norfloxacin starting at 0.016 µM (see below). CEFNOR was included as a control to account for effects of co-dosing with a second antibiotic, in lieu of borrelidin A, that did not demonstrate a CS or CR relationships in the original screen to the Cef^R^ mutants (Liu et al. 2023).

Triplicate cultures were passaged every 24 h, for 12 passages, inoculating a 1:100 dilution per transfer. For populations treated with ceftazidime alone or with borrelidin A, each transfer was conducted under 4 separate 2-fold dilutions of ceftazidime (0.063 to 0.5 µM) for all treatments, and borrelidin A concentrations held constant at either 32 or 128 µM as indicated for CEFBOR32 and CEFBOR128 treatments. For the CEFNOR treatment, four 2-fold dilutions of norfloxacin (0.016 to 0.128 µM) were carried out. For all treatments, OD_600_ absorbance readings were used to determine the highest concentration of ceftazidime resulting in at least 50% growth inhibition for each replicate and used to inoculate the subsequent day's treatment wells. Ceftazidime and norfloxacin drug concentrations for the CEFNOR treatment were modified to ensure that at least one concentration produced 100% growth. Selecting drug concentrations resulting in consistent reduction in growth was done to keep the overall mutational load at a constant rate. Once the appropriate concentrations were determined, inocula were incubated statically at 37 °C, 5% CO_2_ (Liu et al. 2023).

Selection in borrelidin A alone was not performed due to limited availability and lack of antibacterial effects of the compound on its own; borrelidin A was purified in house from marine-derived Actinobacterial cultures (Wong et al. 2012) in relatively low yields, and minimum inhibitory concentrations (MICs) of borrelidin A alone toward the ancestor were >256 µM (Liu et al. 2023). Similarly, limited availability meant that we could not carry out selection under increasing concentrations of borrelidin A.

**Fig. 1.**
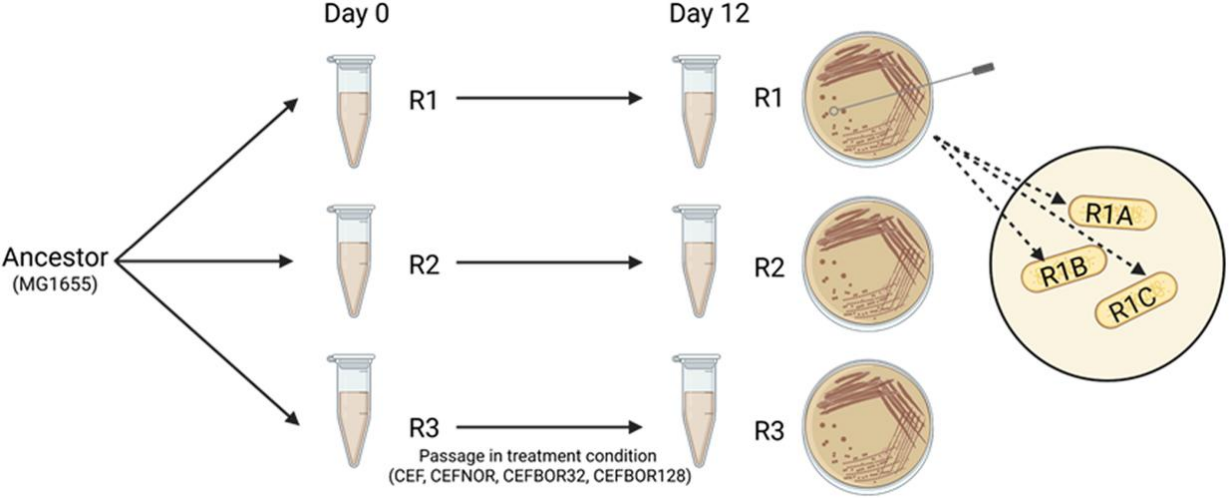
Experimental design for evolved strains from Liu et al. (2023). Ancestral MG1655 *E. coli* was sequentially passaged over 12 d, in triplicate, in each of 4 treatments: (i) CEF: increasing concentrations of ceftazidime starting at 0.063 µM; (ii) CEFNOR: CEF treatment with additional increasing concentrations of norfloxacin starting at 0.016 µM; (iii) CEFBOR32: CEF treatment plus a constant concentration of borrelidin A at 32 µM; or (iv) CEFBOR128: CEF treatment plus a constant concentration of borrelidin A at 128 µM. Replicate cultures (R1, R2, and R3) per treatment were passaged every 24 h for 12 passages, inoculating a 1:100 dilution per transfer. Day 12 populations were streaked onto LBA, and 3 single colonies per replicate (R*#-*A, R*#-*B, and R*#-*C) were used to establish each experimental strain, for a total of 36 strains. Created in BioRender. Phillips, L. (2026) https://BioRender.com/4b2ukw0.

Glycerol stocks of each evolved population were streaked onto LBA, and single colonies were used to establish 3 strains from each of the original evolved populations, for a total of 36 strains (4 treatments × 3 replicate populations per treatment × 3 strains per population) (Fig. 1).

### MIC assays

For each of the evolved strains and their ancestor (MG1655), we used microbroth dilution MIC assays to assess antibiotic susceptibility toward 7 antibiotics: ceftazidime (Cef; in dH_2_O), ampicillin (Amp; in dH_2_O), meropenem (Mer; in dH_2_O), nalidixic acid (Nal; in dH_2_O), norfloxacin (Nor; in DMSO), levofloxacin (Levo; in 0.1 M NaOH), and ciprofloxacin (Cip; in 0.1 M NaOH).

MIC assays were prepared following Hinz et al. (2024) with modifications as follows: 2-fold serial dilutions of antibiotics were prepared in 125 µL of LB broth in 96-well plates, and 125 µL of *E. coli* overnight culture (diluted 1:1000 in LB) were added to each well. Plates were incubated at 37 °C with shaking (150 rpm) for 24 h, with a gas permeable seal. MICs were defined as the lowest antibiotic concentration yielding no visible growth. The following 2-fold concentration ranges were tested: Cef, Nal, and Amp (0.25 to 32 µg/mL) and Cip, Levo, Mer, and Nor (0.015625 to 2 µg/mL). MICs for all evolved strains, as well as MG1655, were performed in duplicate. CEF and CEFNOR strains, along with MG1655, were performed in 1 day, and the CEFBOR32 and CEFBOR128 strains, along with MG1655 again, were performed on a separate instance. CEF replicate R1-C could not be inoculated from glycerol stock and thus was excluded. Fold-change was calculated as the average MIC of each duplicate, compared to the average of the MG1655 duplicates (MIC of the evolved strain divided by the median of the ancestor strain), corresponding to their respective assay.

Statistical analyses were performed in R (version 4.4.0; R Core Team) to assess differences in overall MIC fold-change between the treatment groups for each antibiotic using a Kruskal–Wallis test to assess overall group differences, followed by Dunn's post hoc test for pairwise comparisons with Holm correction for multiple testing using the *dunn.test* package (version 1.3.6).

### Whole-genome sequencing and mutation calling

Genomic DNA was extracted from each of the 36 evolved strains, as well as MG1655, using the Qiagen QIAamp DNA kit according to the manufacturer's instructions. Whole-genome sequencing (WGS) was performed using paired-end 250 bp reads on an Illumina MiSeq at SeqCenter, LLC (Pittsburgh, Pennsylvania). Assembly and mutation calling was carried out using *breseq* (version 0.35.5) (Deatherage and Barrick 2014) set to “consensus mode” on the Galaxy Europe platform (version 25.0.5; https://usegalaxy.eu) (The Galaxy Community 2024) using default parameters. The MG1655 genome assembly was used as a reference (GenBank accession: NC_000913.3). Raw sequence data are available at the European Nucleotide Archive (ENA accession: PRJEB94349).

A 1-way ANOVA was conducted using base R (version 4.4.0; R Core Team 2024) to evaluate treatment effects on average number of observed mutations, followed by a Tukey's Honest Significant Difference (HSD) post hoc pairwise comparison test between treatment groups. Average number of single nucleotide polymorphisms (SNP) and small insertion/deletions (indel), average number of mobile-element insertions (MEI), and the average combined number of mutations were analyzed separately.

### Competitive fitness assays and relative fitness calculations

Relative fitness (*w*) was determined using a co-culture competition assay in LB, against Δ*lac* AH171, in which colonies of evolved strains were blue (Lac+) and the Δ*lac* common competitor white (Lac−) when plated on LBA containing 5-bromo-4-chloro-3-indolyl-β-D-galactopyranoside (X-gal) and isopropyl β-D-1-thiogalactopyranoside (IPTG). A control competition between the ancestor MG1655 and AH171 was used to account for fitness effects of the *lac* operon knockout. Four replicate competitions, derived from independently inoculated cultures, were performed for each evolved strain, as well as ancestral MG1655. Cultures were prepared in 24-well microplates by inoculating 1.5 mL of LB with a colony from a freshly streaked plate. One colony of AH171 was inoculated into 5 mL of LB and was used as the common competitor in all cases. Starting cultures were incubated at 37 °C with shaking (150 rpm) for 24 h, with a gas permeable seal. Competition mixtures consisted of 50 µL of each evolved strain along with 50 µL of the Δ*lac* AH171, except the case of the 3 CEFNOR-R1 replicates (R1-A, R1-B, and R1-C), where 90 µL of CEFNOR strains were inoculated with 10 µL of the Δ*lac* AH171. Fifteen microliters of each competition mixture was then diluted 1:100 into 1.5 mL of LB media in 24-well microplates and incubated at 37 °C with shaking (150 rpm) for 24 h, with a gas permeable seal. The remaining starting competition mixture was stored as a 25% glycerol stock at −80 °C for later plating (T = 0). After 24 h, inoculated competition mixtures were also stored as a 25% glycerol stock at −80 °C for later plating (T = 24).

Spot plating of both the starting (*i,* T = 0) and competed (*f,* T = 24) cultures were utilized to count numbers of blue (Lac+ evolved strains and MG1655) vs white (Δ*lac* Lac− AH171) colonies on LBA supplemented with 40 µg/mL X-Gal (from 20 mg/mL solution in DMSO) and 1 mM IPTG (from 100 mM solution in dH_2_O).

Relative fitness (*w*) was calculated from the initial (*i*, T = 0) and final (*f*, T = 24) counts of the evolved strains and Lac+ MG1655 (n1) and the Δ*lac* AH171 (n2) common competitor following the equation derived by Dykhuizen and Hartl (1983) (Melnyk et al. 2015):


(Eq. 1) $$w=1+\;\frac{\ln\;\left(\frac{n1_{f}}{n1_{i}}\right)-ln\left(\frac{n2_{f}}{n2_{i}}\right)}{No.\;of\;generations}$$

The number of generations was inferred from the 1:100 dilution of the competition mixture, calculated as log_2_(100) = 6.6. Average fitness was calculated as the average fitness of the 4 competition replicates per strain. An overall average fitness was also calculated per treatment group as the average of the nine fitness values per treatment. Relative fitness (*w*) values > 1 are indicative of greater relative fitness than the common competitor, while values < 1 are indicative of lower relative fitness than the common competitor.

A nested 2-way ANOVA was conducted using base R (version 4.4.0; R Core Team 2024) to evaluate treatment effects on the differences in relative fitness between evolved strains, as well as the ancestor, followed by a Tukey's HSD post hoc pairwise comparison test between treatment groups.

## Results

### Borrelidin A co-dosing decreases overall resistance to multiple antibiotics

**Fig. 2.**
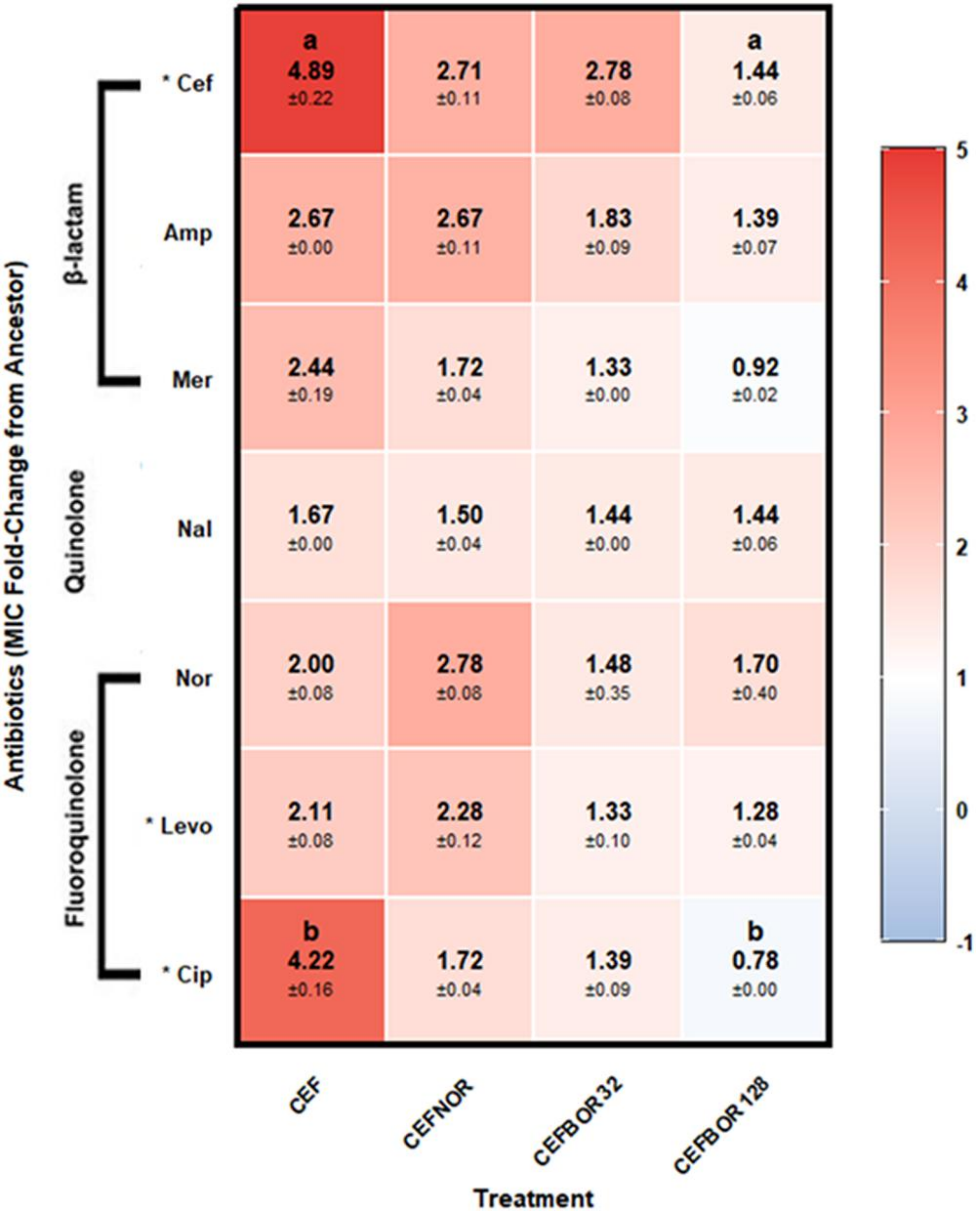
Co-dosing ceftazidime and borrelidin A decreases overall resistance to multiple antibiotics. Average MIC fold-change from ancestral MG1655 *E. coli* for all 9 strains per treatment (CEF, CEFNOR, CEFBOR32, and CEFBOR128), tested against 3 β-lactam antibiotics (ceftazidime [Cef]; ampicillin [Amp]; meropenem [Mer]), 1 quinolone antibiotic (nalidixic acid [Nal]), and 3 fluoroquinolone antibiotics (norfloxacin [Nor]; levofloxacin [Levo]; ciprofloxacin [Cip]). CEF- and CEFNOR-evolved treatment strains consistently have greater levels or resistance compared to CEFBOR32- and CEFBOR128-evolved treatment strains across each tested antibiotic. ±Standard error of the mean (SEM). CR, cross-resistance (>1); CS, collateral sensitivity (<1). “*” indicates antibiotic environment with significant difference (*P* < 0.05) between treatments; “a” significant difference between CEF and CEFBOR128 treatment to Cef resistance; “b” significant difference between CEF and CEFBOR128 treatment to Cip resistance (Supplementary Table 1).

We previously showed that co-dosing of ceftazidime and borrelidin A suppressed the evolution of ceftazidime resistance at the population level (Liu et al. 2023). To further investigate the effects of co-dosing on CS and CR, we isolated single colonies from each experimental population from Liu et al. (2023) and performed MIC assays to assess changes in susceptibility and resistance to additional antibiotics (Fig. 2). MICs included all 9 strains per treatment (as described in Fig. 1 from Methods), expressed as a fold-change from the ancestral strain MG1655 to 3 β-lactam antibiotics (Cef, Amp, and Mer), 1 quinolone (Nal), and 3 fluoroquinolones (Nor, Levo, and Cip). Overall, we hypothesized that co-dosing of ceftazidime and borrelidin A would suppress the evolution of ceftazidime resistance and impede CRs to additional antibiotics as an indirect effect.

CEF evolved strains had the greatest increase in resistance to ceftazidime (4.89-fold increase [±0.22 SEM]) as expected, and in agreement with the populations from Liu et al. (2023). CEF strains also demonstrated CR to all antibiotics tested, ranging from a 1.67-fold increase (±0.00 SEM) toward nalidixic acid to a 4.22-fold increase (±0.16 SEM) toward ciprofloxacin. Co-dosing with ceftazidime and 128 µM borrelidin A resulted in a significant reduction in resistance toward both ceftazidime (Kruskal–Wallis: χ^2^ = 9.43, df = 3, *P* = 0.024; Dunn's post hoc with Holm correction: *P* = 0.007; Supplementary Table 1) and ciprofloxacin (Kruskal–Wallis: χ^2^ = 9.01, df = 3, *P* = 0.029; Dunn's post hoc with Holm correction: *P* = 0.011; Supplementary Table 1), compared to ceftazidime treatment alone. Co-dosing with 32 µM borrelidin A also reduced resistance to both ceftazidime and ciprofloxacin, although the difference was not significant (Supplementary Table 1).

The CEFNOR treatment was used as a control for the effects of a secondary antibiotic, in lieu of borrelidin A, where norfloxacin demonstrated neither CS nor CR with the Cef^R^ strains in our previous work (Liu et al. 2023). CEFNOR-evolved strains demonstrated CR to all tested antibiotics, indicating that co-dosing ceftazidime with norfloxacin did not impede the evolution of CR. In general, strains evolved in CEFNOR had an increase in resistance as compared to CEF evolved strains, but the difference was nonsignificant in all cases. CEFNOR strains did demonstrate increased resistance to norfloxacin (2.78-fold increase [±0.08 SEM]) as compared to CEF (2.00-fold increase [±0.08 SEM]), and additionally had increased CR to levofloxacin, another fluoroquinolone (2.28-fold increase [±0.12 SEM]) as compared to CEF evolved strains (2.11-fold increase [±0.08 SEM]). These differences were again nonsignificant.

Overall, co-dosing of ceftazidime and borrelidin A suppressed the evolution of ceftazidime resistance, as well as CR, and in some cases led to CS to additional antibiotics.

### Co-dosing of ceftazidime and borrelidin A selects against known resistance mutations

In our original study, borrelidin A showed antibacterial activity against ceftazidime resistant strains with mutations in *rfaH* or *rfaG* (Liu et al. 2023) (Supplementary Table 2), both of which are involved in lipopolysaccharide (LPS) biosynthesis (Pagnout et al. 2019; Wang et al. 2021). We therefore hypothesized that borrelidin A would select against *rfa* mutations during laboratory evolution. A secondary screen additionally found borrelidin A activity against strains carrying mutations in *gyrA*, *envZ*, *cyoA*, *ubiF*, and *rpsL*, which may also therefore be selected against (Liu et al. 2023) (Supplementary Table 2). Thus, we used WGS to investigate the genetic basis of resistance in the evolved strains. On average, WGS gave 512,805 reads per strain (190,071 to 815,773; SEM ± 27,444), with an average mapped reads of 92.2% (81.2% to 94.3%; SEM ± 0.4%), and mean coverage of 26.5X (8.3 to 40.4; SEM ± 1.0) (Supplementary Table 3). Replicate CEFBOR32-R2-B had substantially fewer reads, impacting the mutation calls by *breseq.*

**Fig. 3.**
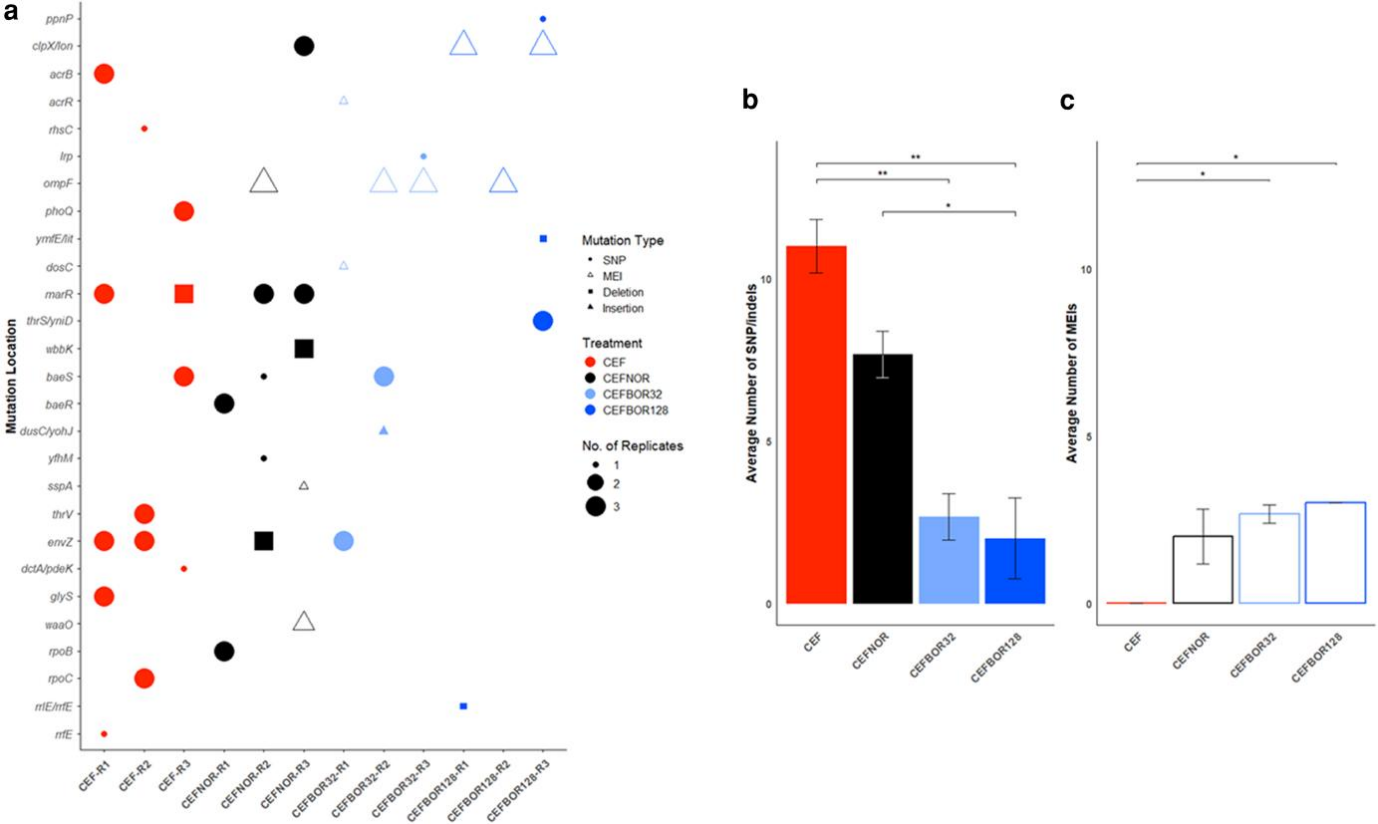
Co-dosing ceftazidime and borrelidin A selects against known resistance mutations. a) Gene name where mutation was observed (mutation location), or intergenic region between 2 genes (gene names separated by “/”), per treatment replicate (CEF, CEFNOR, CEFBOR32, and CEFBOR128). Bubble shape indicates type of mutation: circle = single nucleotide polymorphism (SNP), square = deletion, filled triangle = insertion, open triangle = MEI. Bubble size indicates number of replicate strains with associated mutation. b) Average number of SNP and indel mutations per treatment condition and c) average number of MEI mutations per treatment condition given all nine treatment strains (CEF, CEFNOR, CEFBOR128, and CEFBOR128), ±standard error of the mean (SEM). One-way ANOVA with Tukey’s HSD post hoc significance codes: *P* ≤ 0.001 “***”, 0.01 “**”, and 0.05 “*”. See Supplementary Table 4 for mutation details and Supplementary Tables 5 (SNP and indel), 6 (MEI), and 7 (SNP, indel and MEI combined) for ANOVA results.

Numerous SNP and indel mutations in genes associated with antibiotic resistance and cell permeability were observed in the CEF and CEFNOR strains, including *marR* (multiple antibiotic resistance efflux; CEF-R1 and R3 and CEFNOR-R2 and R3), *acrB* (multidrug efflux pump; CEF-R1), *baeS* (CEF-R3 and CEFNOR-R2-B) and *baeR* (CEFNOR-R1) (efflux regulators), and *envZ* (outer membrane diffusion pore regulatory sensor; CEF-R1 and R2 and CEFNOR-R2). Additionally, mutations in *rpoB* (CEFNOR-R1) and *rpoC* (CEF-R2) encoding the RNA polymerase β and β′ subunits, respectively, were identified and are both known targets for rifamycin resistance (Goldstein 2014) and are associated with general stress responses (Xiao et al. 2017; Nandy et al. 2020). While we expected that the CEF- and CEFNOR-evolved strains would likely contain *rfaH* or *rfaG* mutations conferring ceftazidime resistance (Liu et al. 2023), only a related gene *waaO* (*rfaI*) was found to contain a MEI mutation in 2 CEFNOR replicates (R3-A and C). No *rfaH* or *rfaG* mutations were observed in any evolved strain (Fig. 3a).

Under the borrelidin A treatments, by contrast, we saw very few SNP and indel mutations in known resistance genes. Under 128 µM borrelidin A, we saw no SNP and indel mutations in any of the efflux or porin-related genes that were prevalent under CEF or CEFNOR (Fig. 3a). Under 32 µM borrelidin A, we did observe mutations in *envZ* (CEFBOR32-R1) and in *baeS* (CEFBOR32-R2-A and R2-C), but not in any other known resistance genes.

We did observe several MEIs in the norfloxacin and borrelidin A–treated strains (Fig. 3a). In addition to the CEFNOR MEI found in *waaO*, CEFNOR-R2, CEFBOR32-R2 and R3, and CEFBOR128-R3 all contained MEIs in the outer membrane porin F gene, *ompF*. CEFBOR32-R1-B also notably contains an MEI in the efflux regulator gene, *acrR*. CEFBOR128-R1 and R3 further notably contain MEIs in the intergenic region between the genes *clpX* and *lon*.

Differences in mutational profile are also reflected in overall mutation counts. For SNP and indel mutations, CEF evolved strains had the greatest number of mutations with an average of 11 (±0.82 SEM) per strain, followed by CEFNOR with an average of 7.67 (±0.72 SEM). CEFBOR32 evolved strains only had an average of 2.67 (±0.72 SEM) mutations per strain, while CEFBOR128 strains only had an average of 2 (±1.23 SEM) mutations each. Overall, treatment had a significant effect on the number SNP and indel of mutations (1-way ANOVA, *P* = 0.001; Supplementary Table 5), and co-dosing with borrelidin A generally led to lower numbers of such mutations (Fig. 3b).

Co-dosing with borrelidin A, however, significantly increased the average number of observed MEI mutations (1-way ANOVA, *P* = 0.015; Supplementary Table 6) in comparison to CEF alone (Fig. 3c). CEF treatment strains contained no MEIs while CEFNOR had an average of 2 (±0.82 SEM), CEFBOR32 an average of 2.67 (±0.27 SEM), and CEFBOR128 an average of 3 (±0.00 SEM). When accounting for total number of mutations (SNP and indels combined with MEI), there is a small significant difference in the average number of mutations, but no post hoc combination of treatment conditions was significant (Supplementary Table 7).

### Borrelidin A co-dosing has no negative overall relative fitness effects

**Fig. 4.**
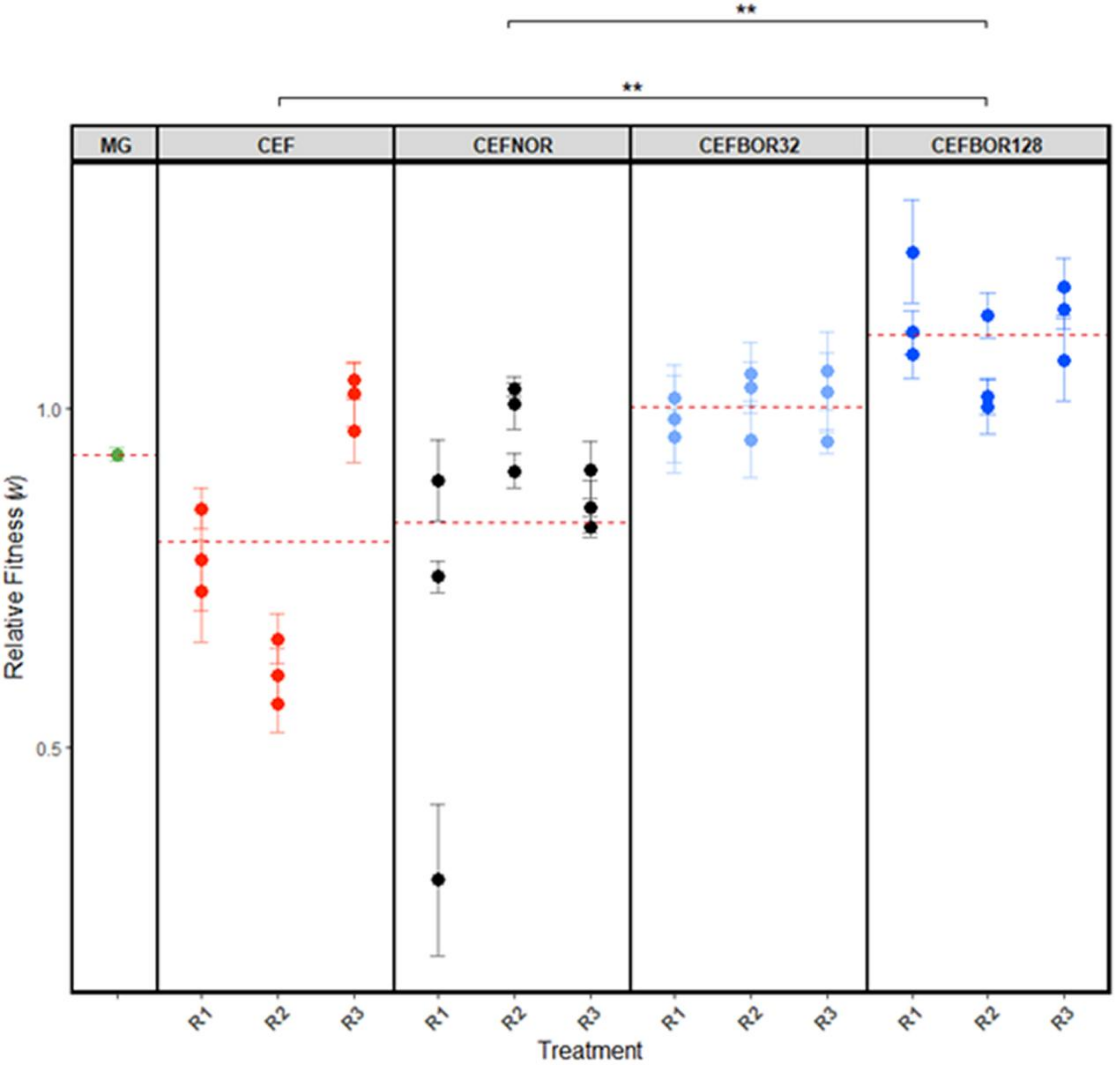
Selection under ceftazidime and borrelidin A co-dosing does not result in a reduction in fitness. Relative fitness (*w*) of ancestral *E. coli* (MG), and evolved strains for each treatment, selected in ceftazidime alone (CEF), ceftazidime and norfloxacin (CEFNOR), ceftazidime and 32 µM borrelidin A (CEFBOR32), or ceftazidime and 128 µM borrelidin A (CEFBOR128). Each data point shows mean of 4 replicates per strain; error bars denote standard error of the mean (SEM); red dotted line denoted the average of the 9 strains per treatment. Two-way ANOVA with Tukey HSD post hoc significance codes: *P* ≤ 0.001 “***”, 0.01 “**”, and 0.05 “*” (Supplementary Table 8).

Lastly, we investigated whether co-dosing of ceftazidime and borrelidin A conferred competitive fitness costs in antibiotic-free environments (Fig. 4). AMR mutations are generally expected to be costly (Melnyk et al. 2015; Chowdhury and Findlay 2023); however, the fitness effects of co-dosing ceftazidime and borrelidin A were unknown. Relative fitness (*w*) of each treatment strain, as well as the ancestor MG1655, was assayed using a competition assay in rich LB media. Fitness effects were estimated by calculating fitness (*w*) for each strain relative to a Δ*lac* strain AH171 (Eq. 1).

Average *w* for both the CEF- and CEFNOR-evolved strains were below that of the ancestor MG1655 at 0.80 (±0.02 SEM) and 0.83 (±0.02 SEM), respectively. However, this loss in fitness was not significant (2-way ANOVA: df = 4, *P* = 0.001; Tukey’s HSD: *P* = 0.922 and *P* = 0.968, respectively; Supplementary Table 8). The average *w* for co-dosed strains was greater than the ancestor at 1.00 (±0.02 SEM) and 1.11 (±0.01 SEM), for CEFBOR32 and CEFBOR128, respectively, but again these differences were not significant (2-way ANOVA: df = 4, *P* = 0.001; Tukey’s HSD: *P* = 0.991 and *P* = 0.797, respectively; Supplementary Table 8); average ancestor *w* was 0.93 (±0.01 SEM). CEFBOR128-evolved strains did however have a significantly higher average *w* compared to the CEF and CEFNOR strains (2-way ANOVA: df = 4, *P* = 0.001; Tukey’s HSD: *P* = 0.002 and *P* = 0.005, respectively; Supplementary Table 8). These results indicate that populations selected under a ceftazidime-borrelidin A co-dosing regimen do not suffer fitness costs as compared to the ancestor but do have a higher fitness as compared to mono-dosed ceftazidime treatment harboring more known resistance mutations.

## Discussion

CS represents an evolution-aware approach to slow the emergence of AMR (Imamovic and Sommer 2013; Lázár et al. 2013; Maltas et al. 2025), whereby combinations of therapeutics are deployed to limit, prevent, or reverse the evolution of AMR. Here, we further the understanding of a novel CS relationship identified in Liu et al. (2023) between ceftazidime and the natural product borrelidin A.

In our original study, borrelidin A showed increased antibacterial activity against ceftazidime resistant mutants carrying mutations in *rfaG* or *rfaH* (Supplementary Table 2). Both *rfaG* and *rfaH* contribute to LPS assembly (Leeds and Welch 1996; Pagnout et al. 2019); *rfaG* encodes the LPS core biosynthesis protein enzyme RfaG (Austin et al. 1990), and *rfaH* encodes a transcription antiterminator (Leeds and Welch 1996). Populations of *E. coli* evolved in the presence of both ceftazidime and borrelidin A achieved lower levels of Cef resistance than did populations evolved in Cef alone or in a combination of Cef and Nor (Liu et al. 2023). We therefore hypothesized that borrelidin A selects against *rfa* mutations in the presence of ceftazidime.

While no de novo *rfaG* or *rfaH* mutations were observed in any experimental strains, a related *waaO* (*rfaI*) MEI mutation was found under CEFNOR conditions. *waaO* is a member of the same *waa* (*rfa*) operon as *rfaG*, encoding the glycosyltransferase that adds glucose residues to the LPS outer core (Pagnout et al. 2019). No *rfa* (*waa*) mutations were observed when borrelidin A was present.

CEF- and CEFNOR-evolved strains did contain a variety of mutations in well characterized antibiotic resistance genes (Fig. 3a; Supplementary Table 4), including those involved in drug efflux such as *marR* (Cohen et al. 1993), *acrB* (Adler et al. 2016), *baeS* and *baeR* (Nagasawa et al. 1993), as well as the outer membrane diffusion pore sensor *envZ* (Adler et al. 2016). These mutations are not observed in CEFBOR strains. The original CS profiling (CSP) screen by Liu et al. (2023) also included multiple mutants sensitive to borrelidin A with mutations in some of the genes mentioned above: R77H and H120fs *marR* mutants, E118* and A151fs *acrB* mutants, and T402M and P248S *envZ* mutants (Supplementary Table 2), suggesting that borrelidin A likely selected against these resistance mutations. Additionally, multiple *rpoB* mutants (I572L, I572S, and D516N) were originally identified as CS to borrelidin A in the CSP (Supplementary Table 2); here, *rpoB* mutants were found in CEFNOR and related mutants in CEF, yet no such mutants were found in borrelidin A co-dosed strains (Fig. 3a).

To ensure changes in mutational profiles were minimally impacted by population size (and in turn mutational load), concentrations of ceftazidime (and norfloxacin in the case of CEFNOR strains) during laboratory selection were adjusted such that the highest drug concentration resulting in a 50% growth inhibition was always used. In the case of CEFNOR strains, the concentrations were further chosen such that at least one of the drug concentrations produced 100% growth. Population size was thus kept fairly consistent between treatments, so differences in mutation profile are unlikely to be due to variation in mutational input.

Overall, strains co-dosed with Cef and borrelidin A had decreased resistance to all tested antibiotics (Fig. 2), which can broadly be attributed to the presence of fewer resistance mutations. The total number of SNP and indel mutations was significantly reduced in CEFBOR evolved strains compared to CEF and CEFNOR (Fig. 3b). Populations selected at 32 µM borrelidin A did show some evidence of efflux and permeability-mediated resistance, with *envZ* mutations in CEFBOR32-R1 and *baeS* mutations in CEFBOR32-R2 strains. However, populations co-dosed at 128 µM borrelidin A saw no point mutations or small indels in these genes.

CEFBOR strains, interestingly, had a significant increase in the prevalence of MEI mutations (Fig. 3c). CEFBOR32-R2 and R3, as well as CEFBOR128-R2 all contain the same MEI (IS*5*) in the *ompF* porin gene. CEFNOR-R2 strains contained a similar MEI (IS*3*) in a different location within the gene. It is likely that any CR and CS observed in the CEFBOR strains would be attributed in part to this *ompF* insertion mutation, with reduced permeability through the porin channel, decreasing overall intracellular accumulation of the drug (Choi and Lee 2019). Additionally, CEFBOR128-R1 and CEFBOR128-R3 contain a MEI (IS*186*) in the intergenic region between the *clpX* and *lon* genes. ClpX is an ATP-dependent protease known to participate in stress responses (Gottesman et al. 1993; Flynn et al. 2004; Neher et al. 2006) and has previously been associated with ampicillin and ciprofloxacin tolerance (Deter et al. 2020).

While an *ompF* MEI was observed in CEFNOR-R2, the overall trend of more MEIs in CEFBOR suggest that borrelidin A does not select against such MEIs. Given that we do observe low levels of resistance to most tested antibiotics in the CEFBOR strains (Fig. 2), it can be deduced that these mutations do confer some resistance, but are out competed in the absence of borrelidin A, where more strongly resistance-conferring SNP and indel AMR mutations prosper; we propose that MEI mutations are maintained in populations when borrelidin A is present due to selection against these larger-effect point and indel mutations.

Given the differences in mutation profile between treatments, we investigated the fitness impacts of co-dosing ceftazidime and borrelidin A. While our data suggest that co-dosing selects against many resistance mutations, the overall effect of co-dosing of CS drugs on fitness was previously unknown. Resistance mutations are generally costly in drug-free environments, resulting in reduced relative fitness compared to nonresistant strains (Melnyk et al. 2015; Vogwill and MacLean 2015). Since co-dosing selects against many resistance mutations, we therefore might expect lower fitness costs.

We found no significant change in relative fitness of both borrelidin A co-dosed treatments compared to the ancestor (Fig. 4; Supplementary Table 8). CEFBOR128 co-dosed strains did however have significantly higher fitness than those evolved in CEF alone or CEFNOR. This observation may be practically beneficial, as it suggests that co-dosing may allow higher-fitness strains lacking major resistance mutations to outcompete strains that confer stronger resistance but incur fitness costs (Hinz et al. 2024), thereby limiting continued selection for higher resistance over time.

For example, CEF and CEFNOR strains contained mutations involved in membrane permeability (ex., *acrB*, *marR*, *baeSR*), where increased energetic burdens required for upregulation of efflux systems may negatively impact overall relative fitness. We suspect that selection against these resistance mutations by borrelidin A also eliminated their associated costs (Melnyk et al. 2015). More broadly, the impacts of CS on fitness are largely unknown, with both overall increases and decreases of relative fitness being observed, even among mutants of the same affected gene (Barbosa et al. 2017; Nichol et al. 2019), highlighting the unpredictability of the relationship.

Both CEFBOR treatment conditions resulted in elevated relative fitness compared to the ancestor, albeit nonsignificantly. Disruption of *ompF* by MEIs in these strains could confer a slight advantage in competition due to compensatory expression of other porins for nutrient resources, such as OmpC and PhoE (Nikaido 1992; Knopp and Andersson 2015), or, speculatively, via reduced permeability of waste by-products in competition; experimental validation would be required to confirm these hypotheses. We observed the highest relative fitness in CEFBOR128-R1 and R3 (average *w* = 1.141 ± 0.030 SEM and 1.133 ± 0.027 SEM, respectively compared to 0.932 ± 0.010 SEM for MG1655), both of which contained a MEI in the intergenic region between the *clpX* and *lon* genes. As noted above, *clpX* is known to participate in stress response signaling and may therefore confer an advantage under competition (Gottesman et al. 1993; Flynn et al. 2004; Neher et al. 2006).

All 3 CEFBOR128-R3 strains had a G→A SNP at position 1,802,589, in the intergenic region between *thrS* and *yniD.* This mutation is likely located in the 5′UTR of the *thrS* mRNA gene, the known target of borrelidin A (Li et al. 2014; Liu et al. 2023). The 5′UTR region of the *thrS* gene forms an operator with similar structure to the threonyl-tRNA anticodon, working as a negative autoregulator of the ThrS operon (Springer et al. 1988). We thus hypothesize that this SNP is a resistance mutation for borrelidin A. Since borrelidin A binds to the threonyl-tRNA synthetase, we propose that mutations in the operator reduce the negative autoregulation, allowing for increased translation of the threonyl-tRNA synthetase enzyme (Springer et al. 1988), overcoming inhibition by borrelidin A. Further validation of this hypothesis will be the subject of future investigation. Identification of this SNP does highlight that bacteria can still evolve toward resistance under CS selective pressures.

This study serves as an example of de novo mutation accumulation under CS conditions. Currently, most studies on the evolution of resistance under CS start with known resistance mutants and track their trajectory during laboratory selection (Imamovic and Sommer 2013; Lázár et al. 2013; Barbosa et al. 2017; Wang et al. 2025), or simulate evolutionary paths with cyclical CS dosing (Nichol et al. 2019). Since resistance will often evolve through multiple pathways (Baquero et al. 2021), *de novo* selection experiments offer a more complete picture of the impact of CS. For example, in the case of ceftazidime, resistance can evolve mutations in multiple genes such as *rfaG*, *rfaH* (Liu et al. 2023), *acrR*, *marR*, *sdiA* (Tavío et al. 2014), and *ampC* (Ogawa et al. 2025) and by horizontally transferred β-lactamases (Bush 2018). For CS to be applicable clinically, compounds should select against a range of resistance mechanisms, as is apparently the case for borrelidin A. We have therefore offered insight into the mechanisms behind CS starting from a susceptible ancestor, to investigate alternate evolutionary paths, and ultimately suppression of evolved resistance.

The ability of borrelidin A to select against a broad spectrum of resistance mechanisms might recommend its use as an adjuvant for the prevention of ceftazidime resistance. Unfortunately, borrelidin A is known to be cytotoxic (Berger et al. 1949; Ishiyama et al. 2011), likely due to its potent threonyl-aminoacyl tRNA synthetase (threonine-tRNA ligase) inhibition both in prokaryotes and eukaryotes (Nass and Hasenbank 1970; Paetz and Nass 1973). This cytotoxicity precludes borrelidin A's clinical use, but compounds of similar nature to borrelidin A have shown significant promise against other microbes. For example, the benzoxaborole compound AN2690 (tavaborole) is a leucyl-tRNA synthetase (LeuRS) inhibitor with FDA approval for the treatment of fungal onychomycosis (Rock et al. 2007; Zhang and Ma 2019), and derivatives have shown effectiveness against *S. pneumoniae* (Hu et al. 2013). Further studies have identified and designed additional compounds which inhibit LeuRS (Zhang and Ma 2019), as well as compounds that target ThrRS with greater bacterial specificity (Guo et al. 2020). Future experiments should therefore investigate co-dosing of compounds including additional aminoacyl-tRNA synthetase (aaRS) inhibitors in combination with currently available antibiotics in a CS manner for the possibility of resensitizing AMR mutants or inhibiting the evolution of drug and multidrug resistance.

## Supplementary Material

jkag046_Supplementary_Data

## Data Availability

Strains are available upon request. Supplementary File S1 contains Supplementary Table 1 describing the complete Kruskal–Wallis and Dunn's post hoc statistics for MICs; Supplementary Table 2 describing collateral sensitivity profiling drug-resistant strains of *E. coli*; Supplementary Table 3 WGS sequencing and coverage metrics; Supplementary Table 4 describing WGS mutation profiles; Supplementary Tables 5 to 7 describing ANOVA and Tukey HSD post hoc statistics for numbers of SNP and indels, MEIs, and combined numbers of mutations, respectively; and Supplementary Table 8 describing ANOVA and Tukey HSD post hoc statistics for relative fitnesses. All raw data have been deposited in the GSA Figshare portal (https://doi.org/10.25387/g3.29630732), including raw MIC values, raw competition counts, and *breseq* mutation outputs. Sequence data are available at the European Nucleotide Archive (ENA) with the accession number PRJEB94349. Supplemental material available at G3 online.
