# Supplementary material for: Mutational basis of ceftazidime-borrelidin A collateral sensitivity in Escherichia coli
Source: G3 (Bethesda). 2026 Mar 4;16(5):jkag046. doi: 10.1093/g3journal/jkag046 (PMC13148393; doi:10.1093/g3journal/jkag046)
Supplement: jkag046_Supplementary_Data [file jkag046_supplementary_data.docx]

**Supplemental Materials:**

**Supplemental Table 1:** Complete Kruskal-Wallis test (one-way ANOVA on ranks for non-parametric statistical tests) and Dunn’s post-hoc with Holm adjustment, for changes in minimum inhibitory concentration (MIC) compared to ancestral *E. coli* MG1655 of CEF, CEFNOR, CEFBOR32, and CEFBOR128 evolved strains tested against three β-lactam antibiotics (ceftazidime [Cef]; ampicillin [Amp]; meropenem [Mer]), one quinolone antibiotic (nalidixic acid [Nal]), and three fluoroquinolone antibiotics (norfloxacin [Nor]; levofloxacin [Levo]; ciprofloxacin [Cip]). Significant *p*-values in **bold** (α < 0.05 for ANOVA; α < 0.05/2 for Dunn’s post-hoc).

| **Kruskal-Wallis** |  | **Cef** | **Amp** | **Mer** | **Nal** | **Nor** | **Levo** | **Cip** |
| --- | --- | --- | --- | --- | --- | --- | --- | --- |
| *df* = 3 | *χ^2^* | 9.425 | 6.735 | 6.885 | 1.084 | 5.408 | 8.897 | 9.011 |
|  | *p*-value | **0.024** | 0.081 | 0.076 | 0.781 | 0.144 | **0.031** | **0.029** |
| **Dunn’s post-hoc** |  |  |  |  |  |  |  |  |
| CEF-CEFNOR | *Z* | 1.591 | -0.228 | 0.345 | 0.521 | -1.144 | -0.573 | 1.140 |
|  | Raw *p* | 0.223 | 0.410 | 0.365 | 1.000 | 0.505 | 0.567 | 0.381 |
| CEF-CEFBOR32 | *Z* | 1.477 | 1.140 | 1.148 | 0.868 | 1.087 | 1.892 | 1.88 |
|  | Raw *p* | 0.210 | 0.381 | 0.376 | 0.964 | 0.416 | 0.117 | 0.150 |
| CEF-CEFBOR128 | *Z* | 3.068 | 2.053 | 2.412 | 0.925 | 0.515 | 1.662 | 2.908 |
|  | Raw *p* | **0.007** | 0.100 | 0.048 | 1.000 | 0.303 | 0.145 | **0.011** |
| CEFNOR-CEFBOR32 | *Z* | 0.114 | -1.368 | -0.804 | -0.347 | -2.231 | -2.465 | -0.741 |
|  | Raw *p* | 0.455 | 0.342 | 0.421 | 0.729 | 0.077 | 0.041 | 0.229 |
| CEFNOR-CEFBOR128 | *Z* | -1.477 | -2.281 | -2.067 | -0.405 | -1.659 | -2.235 | -1.767 |
|  | Raw *p* | 0.140 | 0.068 | 0.097 | 1.000 | 0.243 | 0.064 | 0.154 |
| CEFBOR32-CEFBOR128 | *Z* | -1.591 | -0.912 | -1.263 | -0.058 | 0.572 | 0.229 | -1.027 |
|  | Raw *p* | 0.279 | 0.362 | 0.413 | 0.477 | 0.567 | 0.409 | 0.305 |

**Supplemental Table 2:** Collateral Sensitivity Profiling drug-resistant *E. coli* strains demonstrating collateral sensitivity to RLUS-1732D (borrelidin A) during primary and/or secondary screening. Cef6 and Cef7 (**bold**) were initial strains of interest; strains in *italics* contain target gene mutation observed in WGS; other strains identified in secondary screen. Δ% growth (PS): change in % growth (OD_600_) from wild-type from Primary Screen (PS); ΔLog_2_(MIC) (SS): Log2(MIC) fold-change from wild-type from Secondary Screen (SS). Modified from Liu et al. (2023).

| **Strain** | **Gene** | **Mutation** | **Δ% growth (PS)** | **ΔLog_2_(MIC) (SS)** | **Protein** | **Selection Antibiotic** | **Drug mechanism** |
| --- | --- | --- | --- | --- | --- | --- | --- |
| **Cef6** | *rfaH* | W4* |  | -3 | Transcription antitermination protein RfaH | Ceftazidime | Cell wall biosynthesis |
| **Cef7** | *rfaG* | E289fs |  | -3 | Lipopolysaccharide core biosynthesis protein RfaG |  |  |
| *Cef1* | *envZ* | T402M | -11.61 | -1 | Sensor histidine kinase EnvZ |  |  |
| *Cef8* |  | P248S | -12.32 | -1 |  |  |  |
| *Cip8* | *marR* | R77H | -2.93 |  | Multiple antibiotic resistance protein | Ciprofloxacin | Multidrug resistance |
| *Tet8* |  | H120fs | -11.5 |  |  | Tetracycline |  |
| *Cm2* | *acrR* | E118* | -15.27 |  | HTH-type transcriptional regulatory AcrR | Chloramphenicol |  |
| *Cm3* |  | A151fs | -5.9 |  |  |  |  |
| *Rif1* | *rpoB* | I572L | -32.99 |  | DNA-directed RNA polymerase subunit beta | Rifampicin | RNA polymerase |
| *Rif7* |  | I572S | -17.34 |  |  |  |  |
| *Rif11* |  | D516N | -24.07 |  |  |  |  |
| Cip5 | *gyrA* | D87G | -18.2 | -1 | DNA gyrase subunit A | Ciprofloxacin | DNA gyrase |
| Gn12 | *cyoA* | I127fs | -9.02 | -1 | Cytochrome bo(3) ubiquinol oxidase subunit 2 | Gentamicin | Protein synthesis |
| Gn14 | *ubiF* | D342H | -10.06 | -1 | 3-demethoxyubiquinol 3-hydroxylase |  |  |
| Kn15 |  | Q120* | 6.39 | -1 |  | Kanamycin |  |
| Str1 | *rpsL* | K43R | -27.04 | -1 | 30S ribosomal protein S12 | Streptomycin |  |

**Supplemental Table 3:** *breseq* sequence and coverage parameters for whole-genome sequencing (WGS) of all 36 evolved strains, as well as the ancestor MG1655 (MG).

| **Strain** | **Total reads (#)** | **Mapped reads (%)** | **Fit mean** | **Fit dispersion** |
| --- | --- | --- | --- | --- |
| MG | 497,445 | 92.8 | 24.8 | 1.4 |
| CEF-R1-A | 815,773 | 92.0 | 40.4 | 1.8 |
| CEF-R1-B | 697,117 | 92.7 | 34.7 | 1.8 |
| CEF-R1-C | 464,300 | 93.8 | 23.5 | 1.6 |
| CEF-R2-A | 626,907 | 92.7 | 31.1 | 1.7 |
| CEF-R2-B | 469,563 | 93.9 | 23.0 | 1.5 |
| CEF-R2-C | 470,128 | 94.3 | 23.9 | 1.5 |
| CEF-R3-A | 456,467 | 91.6 | 22.4 | 1.4 |
| CEF-R3-B | 571,929 | 91.4 | 28.1 | 1.4 |
| CEF-R3-C | 440,336 | 93.1 | 22.0 | 1.4 |
| CEFNOR-R1-A | 416,904 | 92.8 | 20.9 | 1.3 |
| CEFNOR-R1-B | 591,249 | 91.4 | 28.7 | 1.4 |
| CEFNOR-R1-C | 643,615 | 91.4 | 31.6 | 1.4 |
| CEFNOR-R2-A | 573,297 | 91.0 | 28.0 | 1.5 |
| CEFNOR-R2-B | 493,575 | 93.4 | 24.8 | 1.4 |
| CEFNOR-R2-C | 514,512 | 91.7 | 25.3 | 1.8 |
| CEFNOR-R3-A | 557,840 | 92.6 | 27.7 | 1.6 |
| CEFNOR-R3-B | 609,895 | 91.0 | 29.8 | 1.5 |
| CEFNOR-R3-C | 497,910 | 92.5 | 24.3 | 1.4 |
| CEFBOR32-R1-A | 566,981 | 93.9 | 27.7 | 1.7 |
| CEFBOR32-R1-B | 626,496 | 91.9 | 31.0 | 1.5 |
| CEFBOR32-R1-C | 563,168 | 94.1 | 27.6 | 1.8 |
| CEFBOR32-R2-A | 592,781 | 92.8 | 29.6 | 1.5 |
| CEFBOR32-R2-B | 190,071 | 81.2 | 8.3 | 1.4 |
| CEFBOR32-R2-C | 461,329 | 93.4 | 23.2 | 1.4 |
| CEFBOR32-R3-A | 421,135 | 94.2 | 21.3 | 1.5 |
| CEFBOR32-R3-B | 523,012 | 93.7 | 26.4 | 1.6 |
| CEFBOR32-R3-C | 660,812 | 92.4 | 31.9 | 1.7 |
| CEFBOR128-R1-A | 778,428 | 90.9 | 38.1 | 1.7 |
| CEFBOR128-R1-B | 603,375 | 93.3 | 29.4 | 1.6 |
| CEFBOR128-R1-C | 391,809 | 93.0 | 19.6 | 1.5 |
| CEFBOR128-R2-A | 503,332 | 92.4 | 23.8 | 1.7 |
| CEFBOR128-R2-B | 385,588 | 90.4 | 17.0 | 1.5 |
| CEFBOR128-R2-C | 474,126 | 91.7 | 21.5 | 1.6 |
| CEFBOR128-R3-A | 630,684 | 92.1 | 30.6 | 1.7 |
| CEFBOR128-R3-B | 579,674 | 92.2 | 28.5 | 1.7 |
| CEFBOR128-R3-C | 578,069 | 92.8 | 28.8 | 1.6 |
| **Average** | 512,805 | 92.2 | 26.5 | 1.6 |

**Supplemental Table 4:** Whole-genome sequencing (WGS) mutation profile for all 36 evolved strains, using *breseq*, against *E. coli* MG1655 reference genome NC_000913.3. Type: SNP = single nucleotide polymorphism; DEL = deletion; INS = insertion; MEI = mobile-element insertion. • indicates replicates (A/B/C) containing the mutation.

| **Strain** | **Position (bp)** | **Gene** | **Annotation** | **Type** | **A** | **B** | **C** | **Sum SNP/indel** | **Sum MEI** |
| --- | --- | --- | --- | --- | --- | --- | --- | --- | --- |
| CEF-R1 | 482,516 | *acrB* ← | S630T (TCC→ACC) | SNP | **•** | **•** | **•** | 3 |  |
|  | 1,619,260 | *marR* → | C47* (TGC→TGA) | SNP | **•** | **•** | **•** | 3 |  |
|  | 3,535,432 | *envZ* ← | R146L (CGC→CTC) | SNP | **•** | **•** | **•** | 3 |  |
|  | 3,723,430 | *glyS* ← | V323A (GTT→GCT) | SNP | **•** | **•** | **•** | 3 |  |
|  | 4,213,050 | *rrfE* → | (C→A); noncoding (11/120 nt) | SNP |  |  | **•** | 1 |  |
| CEF-R2 | 732,478 | *rhsC →* | R966W (CGG→TGG) | SNP | **•** |  |  | 1 |  |
|  | 3,423,643 | *thrV ←* | (G→T); noncoding (13/76 nt) | SNP | **•** | **•** | **•** | 3 |  |
|  | 3,535,612 | *envZ ←* | I86S (ATC→AGC) | SNP | **•** | **•** | **•** | 3 |  |
|  | 4,186,404 | *rpoC →* | R352L (CGT→CTT) | SNP | **•** | **•** | **•** | 3 |  |
| CEF-R3 | 1,188,974 | *phoQ ←* | Y268C (TAC→TGC) | SNP | **•** | **•** | **•** | 3 |  |
|  | 1,619,302 | *marR →* | - AAAGGTATTGTCGGT Δ15 bp; coding (183‑197/435 nt) | DEL | **•** | **•** | **•** | 3 |  |
|  | 2,163,713 | *baeS →* | S280P (TCT→CCT) | SNP | **•** | **•** | **•** | 3 |  |
|  | 3,683,472 | *dctA ← / ← pdeK* | (G→A); intergenic (‑25/+158) | SNP | **•** |  |  | 1 |  |
| Treatment sum | | | | | | | | 33 | |
| CEFNOR-R1 | 2,164,462 | *baeR →* | M63V (ATG→GTG) | SNP | **•** | **•** | **•** | 3 |  |
|  | 4,184,980 | *rpoB →* | R1246C (CGT→TGT) | SNP | **•** | **•** | **•** | 3 |  |
| CEFNOR-R2 | 986,862 | *ompF ←* | IS*1* (+) +9 bp; (113‑121/1089 nt) | MEI | **•** | **•** | **•** |  | 3 |
|  | 1,619,300 | *marR →* | K61E (AAA→GAA) | SNP | **•** | **•** | **•** | 3 |  |
|  | 2,163,770 | *baeS →* | S299P (TCG→CCG) | SNP |  | **•** |  | 1 |  |
|  | 2,652,236 | *yfhM ←* | C18G (TGC→GGC) | SNP |  |  | **•** | 1 |  |
|  | 3,535,201 | *envZ ←* | - ATATGGTTAAAG Δ12 bp; coding (657‑668/1353 nt) | DEL | **•** | **•** | **•** | 3 |  |
| CEFNOR-R3 | 458,778 | *clpX → / → lon* | (T→C); intergenic (+78/‑110) | SNP | **•** | **•** | **•** | 3 |  |
|  | 1,619,451 | *marR →* | C111Y (TGC→TAC) | SNP | **•** | **•** | **•** | 3 |  |
|  | 2,104,077 | *wbbK ←* | (T)_8→7_; coding (433/1119 nt) | DEL | **•** | **•** | **•** | 3 |  |
|  | 3,377,367 | *sspA ←* | IS*2* (+) +5 bp; coding (50‑54/639 nt) | MEI |  | **•** |  |  | 1 |
|  | 3,802,089 | *waaO ←* | IS*5* (–) +4 bp; coding (967‑970/1020 nt) | MEI | **•** |  | **•** |  | 2 |
| Treatment sum | | | | | | | | 29 | |
| CEFBOR32-R1 | 486,025 | *acrR →* | IS*1* (+) +9 bp; coding (265‑273/648 nt) | MEI |  | **•** |  |  | 1 |
|  | 1,566,879 | *dosC ←* | IS*5* (–) +4 bp; coding (259‑262/1383 nt) | MEI |  | **•** |  |  | 1 |
|  | 3,535,432 | *envZ ←* | R146L (CGC→CTC) | SNP | **•** | **•** | **•** | 3 |  |
| CEFBOR32-R2 | 986,937 | *ompF ←* | IS*5* (+) +4 bp; coding (43‑46/1089 nt) | MEI | **•** | **•** | **•** |  | 3 |
|  | 2,163,747 | *baeS →* | L291R (CTG→CGG) | SNP | **•** | **•** | **•** | 3 |  |
|  | 2,230,404 | *dusC ← / → yohJ* | (A)_7→8_; intergenic (‑19/‑220) | INS | **•** |  |  | 1 |  |
| CEFBOR32-R3 | 932,898 | *lrp →* | E102* (GAA→TAA) | SNP |  | **•** |  | 1 |  |
|  | 986,937 | *ompF ←* | IS*5* (+) +4 bp; coding (43‑46/1089 nt) | MEI | **•** | **•** | **•** |  | 3 |
| Treatment sum | | | | | | | | 16 | |
| CEFBOR128-R1 | 458,790 | *clpX → / → lon* | IS*186* (+) +6 bp :: Δ1 bp; intergenic (+90/‑93) | MEI | **•** | **•** | **•** |  | 3 |
|  | 4,212,973 | *rrlE → / → rrfE* | -T Δ1 bp; intergenic (+27/‑67) | DEL |  |  | **•** | 1 |  |
| CEFBOR128-R2 | 986,937 | *ompF ←* | IS*5* (+) +4 bp; coding (43‑46/1089 nt) | MEI | **•** | **•** | **•** |  | 3 |
| CEFBOR128-R3 | 408,300 | *ppnP →* | P42S (CCG→TCG) | SNP |  |  | **•** | 1 |  |
|  | 458,790 | *clpX → / → lon* | IS*186* (–) +6 bp :: Δ1 bp; intergenic (+90/‑93) | MEI | **•** | **•** | **•** |  | 3 |
|  | 1,198,498 | *ymfE ← / → lit* | (AATGATGA)_6→5_; intergenic (‑261/‑190) | DEL |  |  | **•** | 1 |  |
|  | 1,802,589 | *thrS ← / → yniD* | (G→A); intergenic (‑19/‑2576) | SNP | **•** | **•** | **•** | 3 |  |
| Treatment sum | | | | | | | | 15 | |

**Supplemental Table 5:** One-way ANOVA test and TukeyHSD post-hoc, for differences in number of SNP and indel mutations observed by whole-genome sequencing of CEF, CEFNOR, CEFBOR32 and CEFBOR128 evolved strains. Significant *p*-values in **bold** (α = 0.05).

| **One-way ANOVA** | ***df*** | **Sum Sq** | **Mean Sq** | **F value** | **Pr (>F)** | **Significance** |
| --- | --- | --- | --- | --- | --- | --- |
| Treatment | 3 | 164.33 | 54.78 | 14.94 | **0.00121** | ** |
| Residuals | 8 | 29.33 | 3.67 |  |  |  |
| **TukeyHSD** | | **Diff** | **Lwr** | **Upr** | **p adj** | **Significance** |
| CEF-CEFNOR | | -3.33333 | -8.34012 | 1.67345 | 0.22210 |  |
| CEF-CEFBOR32 | | -8.33333 | -13.34012 | -3.32655 | **0.00311** | ** |
| CEF-CEFBOR128 | | -9.00000 | -14.00679 | -3.99321 | **0.00191** | ** |
| CEFNOR-CEFBOR32 | | 5.00000 | -0.00679 | 10.00678 | 0.05031 |  |
| CEFNOR-CEFBOR128 | | 5.66667 | 0.65988 | 10.67345 | **0.02776** | * |
| CEFBOR32-CEFBOR128 | | 0.66667 | -4.34012 | 5.67345 | 0.97230 |  |

Significance codes: p < 0.001 ‘***’, 0.01 ‘**’, 0.05 ‘*’

**Supplemental Table 6:** One-way ANOVA test and TukeyHSD post-hoc, for differences in number of MEI mutations observed by whole-genome sequencing of CEF, CEFNOR, CEFBOR32 and CEFBOR128 evolved strains. Significant *p*-values in **bold** (α = 0.05).

| **One-way ANOVA** | ***df*** | **Sum Sq** | **Mean Sq** | **F value** | **Pr (>F)** | **Significance** |
| --- | --- | --- | --- | --- | --- | --- |
| Treatment | 3 | 16.250 | 5.417 | 6.5 | **0.0154** | * |
| Residuals | 8 | 6.667 | 0.833 |  |  |  |
| **TukeyHSD** | | **Diff** | **Lwr** | **Upr** | **p adj** | **Significance** |
| CEF-CEFNOR | | 2.00000 | -0.38689 | 4.38689 | 0.10400 |  |
| CEF-CEFBOR32 | | 2.66667 | 0.27977 | 5.05356 | **0.02961** | * |
| CEF-CEFBOR128 | | 3.00000 | 0.61311 | 5.38689 | **0.01611** | * |
| CEFNOR-CEFBOR32 | | -0666667 | -3.05356 | 1.72023 | 0.80815 |  |
| CEFNOR-CEFBOR128 | | -1.00000 | -3.38689 | 1.38689 | 0.56471 |  |
| CEFBOR32-CEFBOR128 | | -0.33333 | -2.72023 | 2.05356 | 0.96833 |  |

Significance codes: p < 0.001 ‘***’, 0.01 ‘**’, 0.05 ‘*’

**Supplemental Table 7:** One-way ANOVA test and TukeyHSD post-hoc, for differences in number of SNP, indel, and MEI mutations observed by whole-genome sequencing of CEF, CEFNOR, CEFBOR32 and CEFBOR128 evolved strains. Significant *p*-values in **bold** (α = 0.05).

| **One-way ANOVA** | ***df*** | **Sum Sq** | **Mean Sq** | **F value** | **Pr (>F)** | **Significance** |
| --- | --- | --- | --- | --- | --- | --- |
| Treatment | 3 | 82.92 | 270639 | 4.877 | **0.0325** | * |
| Residuals | 8 | 45.33 | 5.667 |  |  |  |
| **TukeyHSD** | | **Diff** | **Lwr** | **Upr** | **p adj** | **Significance** |
| CEF-CEFNOR | | -1.33333 | -7.55759 | 4.89092 | 0.89960 |  |
| CEF-CEFBOR32 | | -5.66667 | -11.89092 | 0.55759 | 0.07495 |  |
| CEF-CEFBOR128 | | -6.00000 | -12.22425 | 0.22425 | 0.05883 |  |
| CEFNOR-CEFBOR32 | | 4.33333 | -1.89092 | 10.55759 | 0.19488 |  |
| CEFNOR-CEFBOR128 | | 4.66667 | -1.55759 | 10.89092 | 0.15419 |  |
| CEFBOR32-CEFBOR128 | | 0.33333 | -5.89092 | 6.55759 | 0.99805 |  |

Significance codes: p < 0.001 ‘***’, 0.01 ‘**’, 0.05 ‘*’

**Supplemental Table 8:** Two-way ANOVA test and TukeyHSD post-hoc, for relative fitness (*w*) effects of ancestral *E. coli* MG1655 (MG) and CEF, CENFOR, CEFBOR32 and CEFBOR128 evolved strains. Significant *p*-values in **bold** (α = 0.05).

| **Two-way ANOVA** | ***df*** | **Sum Sq** | **Mean Sq** | **F value** | **Pr (>F)** | **Significance** |
| --- | --- | --- | --- | --- | --- | --- |
| Treatment | 4 | 0.5704 | 0.14260 | 6.280 | **0.00132** | ** |
| Population | 2 | 0.0861 | 0.04305 | 1.896 | 0.17199 |  |
| Population:Replicate | 6 | 0.0560 | 0.00934 | 0.411 | 0.86420 |  |
| Residuals | 24 | 0.5449 | 0.2271 |  |  |  |
| **TukeyHSD** | | **Diff** | **Lwr** | **Upr** | ***p* adj** | **Significance** |
| MG-CEF | | 0.12999 | -0.33795 | 0.59792 | 0.92237 |  |
| MG-CEFNOR | | 0.10069 | -0.36724 | 0.56862 | 0.96797 |  |
| MG-CEFBOR32 | | -0.07117 | -0.53910 | 0.39677 | 0.99112 |  |
| MG-CEFBOR128 | | -0.17706 | -0.64499 | 0.29087 | 0.79723 |  |
| CEF-CEFNOR | | 0.02929 | -0.17997 | 0.23856 | 0.99352 |  |
| CEF-CEFBOR32 | | 0.20115 | -0.00811 | 0.41042 | 0.06370 |  |
| CEF-CEFBOR128 | | 0.30705 | 0.09779 | 0.51632 | **0.00198** | ** |
| CEFNOR-CEFBOR32 | | -0.17186 | -0.38112 | 0.03741 | 0.1444 |  |
| CEFNOR-CEFBOR128 | | -0.27776 | -0.48702 | -0.06849 | **0.00542** | ** |
| CEFBOR32-CEFBOR128 | | -0.10590 | -0.31516 | 0.10337 | 0.57795 |  |

Significance codes: *p* < 0.001 ‘***’, 0.01 ‘**’, 0.05 ‘*’
